# How does iron cross the abluminal membrane of the blood–brain barrier

**DOI:** 10.1038/s12276-026-01734-y

**Published:** 2026-06-03

**Authors:** Qian Guo, Tianying Wang, Zhong-Ming Qian, Christopher Qian

**Affiliations:** 1 Mental Health Center Affiliated to Shanghai University School of Medicine, Shanghai, China; 2 Laboratory of Drug Delivery, School of Medicine, Shanghai University, Shanghai, China; 3 National Clinical Research Center for Aging and Medicine, Huashan Hospital, Fudan University, Shanghai, China; 4 School of Biomedical Sciences and Gerald Choa Neuroscience Centre, Faculty of Medicine, The Chinese University of Hong Kong, Shatin, New Territories Hong Kong

**Keywords:** Homeostasis, Blood-brain barrier, Translational research

## Abstract

The process of iron transport across the blood–brain barrier (BBB) includes two transmembrane steps: step 1, iron in circulating blood first passes through the luminal membrane (blood side) of BBB cells; step 2, it then passes through the abluminal membrane of the cell (cerebroside) and eventually enters the brain. Compared with step 1, we know relatively little about the mechanism of step 2. However, a large number of studies conducted in the past two decades since the discovery of ferroportin 1 (Fpn1) in 2000 has greatly enhanced our understanding of this issue. Accumulating evidence suggests that Fpn1 is a key player in step 2 and that Fpn1/hephaestin and/or Fpn1/ceruloplasmin iron export pathways found in the basement membrane of intestinal enterocytes have the same role in step 2. In this Review, we focus on the current understanding of the role of Fpn1 in iron transport across the abluminal membrane of the BBB.

## Fpn1 role in iron transport across blood–brain barrier

The blood–brain barrier is a critical regulator of iron transport, essential for neurological processes and implicated in neurodegenerative diseases. This Review focuses on the role of ferroportin 1 (Fpn1) in iron transport across the abluminal membrane of the blood–brain barrier, a less-understood area compared with the luminal membrane. Fpn1, the only known cellular iron exporter, works with hephaestin and ceruloplasmin to oxidize ferrous iron (Fe^2+^) to ferric iron (Fe^3+^), facilitating its transport into the brain. This process is crucial for maintaining iron homeostasis and preventing oxidative damage. The study highlights the expression of Fpn1, hephaestin, and ceruloplasmin in various brain cells, suggesting their broader role in iron efflux. Future research should explore the dominant pathways under physiological conditions and the potential involvement of amyloid-β precursor protein in iron transport, which could inform therapeutic strategies for neurological diseases.

This summary was initially drafted using artificial intelligence, then revised and fact-checked by the author.

## Introduction

The blood–brain barrier (BBB) is a highly selective semi-permeable boundary that separates blood plasm from the brain and extracellular fluid in the central nervous system (CNS)^1^. The barrier consists of microvascular endothelial cells surrounded by the basement membrane and perivascular astrocytic endfeet, all of which act as a distinct entity^2–4^ and form the neurovascular unit^5,6^. Iron is the most abundant metal in the brain^7–9^ and has a crucial role in maintaining various neurological processes^10–12^, whereas excess iron is a powerful source of oxidative damage to brain cells through free radical formation^13–16^. Iron transport to the brain is critical for these multiple neurological processes^17^, and brain iron status is continuously maintained and tightly regulated at the level of the BBB^18–23^. Therefore, understanding the mechanisms of iron transport across the BBB is not only critical to addressing the effects of iron deficiency on brain development and iron over-accumulation in neurodegenerative diseases^17^ but also has potential implications for the field of drug delivery across the BBB, leading to new ways to increase the amount of drug delivered to the brain, especially for the treatment of brain diseases such as Alzheimer’s and Parkinson’s^1,24–26^.

The entire transport process of iron across the BBB consists of two transmembrane steps, namely, step 1: iron in circulating blood first passes through the luminal membrane (apical, blood side) of BMVECs (brain microvascular endothelial cells), and step 2: iron then passes through the abluminal membrane of cells (basal, brain side) and eventually enters the brain^19,27,28^. Accumulated results show that the Tf/TfR1 pathway is the primary transport pathway of iron across the luminal membrane of MVECs (step 1)^7,29–35^. Research has also shown that H-ferritin (as an iron-transport protein)/Tim-1 (T cell immunoglobulin mucin domain 1, as a H-ferritin receptor)^17,36–41^, Lf/LfR (lactoferrin/lactoferrin receptor)^42–45^, sP97/GPI-P97 (secreted melanotransferrin/glycosylphosphatidylinositol (GPI)-anchored p97)^46–50^, transcytosis of Tf/TfR1 complexes^51^, and non-transferrin-bound iron delivery pathways^7,52,53^ all can transport iron across the luminal membrane of the BBB. A recent study reported a novel mechanism whereby exosomes are also involved in the transport of iron (Tf-ferritin and H-ferritin bound iron) across the human BBB endothelial cells^54^.

In contrast to the mechanism of iron crossing the luminal membrane of the BBB (step 1), relatively little is known about the mechanism by which iron passes through the abluminal membrane of the BBB (step 2). Studies relevant to this critical step of iron crossing the BBB have also only become available relatively recently. In fact, until the discovery of membrane ferroportin 1 (Fpn1) in 2000 (refs. ^55–57^), little was known about how iron passed through the abluminal membrane of the BBB. However, a series of studies conducted in the past two decades since Fpn1 was discovered have greatly enhanced our understanding of this issue. Evidence suggests that Fpn1 is a key player in iron transport across the abluminal membrane of the BBB and that Fpn1/hephaestin (Heph) and/or Fpn1/ceruloplasmin (CP) iron export pathways found in the basement membrane of intestinal enterocytes play the same role in step 2 of iron transport across the BBB. Recent advances in the mechanism of iron transport in step 1 have been reviewed elsewhere^19,40,58–64^. In this Review, we focus on a discussion of the current understanding of the role of Fpn1 in step 2.

## Ferroportin 1 has a key role in iron transport across the abluminal membrane of the BBB

**Fig. 1:**
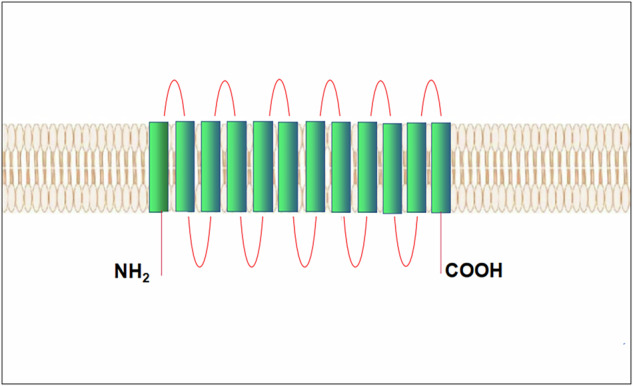
Schematic architecture of ferroportin 1 (Fpn1). It is generally agreed that Fpn1 consists of 12 transmembrane domains with NH_2_ and COOH terminals^65,66^.

Fpn1 (or IREG1//Slc40a1//MTP1) is the only proven cellular iron export protein that is expressed on the basolateral surface of intestinal epithelial cells in mammals. This exporter of cellular iron is directly associated with iron transport across the basolateral membrane of intestinal epithelial cells into circulation and is a key player in the control of iron absorption and iron homeostasis in the body^55–57^. The most recent publicly available data set indicates that this gene encodes a 3330 bp mRNA encoded by eight exons, which is then translated into a transmembrane protein consisting of 571 amino acids^65^. At present, the protein structure is still not fully understood, although experts in the field of iron metabolism generally agree that Fpn1 consists of 12 transmembrane domains^65,66^ (Fig. 1).

The gene is also named Ireg1 (iron regulatory gene) because of the presence of a functional IRE (iron regulatory element) in the 5ʹ-untranslated region of its mRNA^57^. The evidence collected to date suggests that Fpn1 expression or content can be controlled by hepcidin at the post-translational level^67,68^, by the IRE/IRP system at the translational level^69,70^, and by iron and heme at the transcriptional or mRNA level^16,71–73^. Dysregulation of Fpn1 has been implicated in the pathogenesis of iron deficiency anemia and iron overload syndromes including hemochromatosis^56,74–77^.

To date, numerous studies have well established the physiological role of Fpn1 in ferrous iron (Fe^2+^) transport across the basolateral membrane of enterocytes in the intestine by a mechanism that requires an auxiliary ferroxidase activity of Heph and/or CP^55–57,78–80^. In 2001, about a year after Fpn1 was discovered, we proposed that Fpn1 might have the same role in Fe^2+^ transport across the abluminal (or basolateral) membrane of the BBB (step2) as it does in enterocytes^31,81^. A potential role for Fpn1 in step 2 was also proposed later by other research groups^21,82–84^.

After years of accumulated studies on the expression and function of Fpn1 in the brain, it is now well established that Fpn1 mediates the transport of ferrous iron across the abluminal membrane of the BBB^7,85^, just as it does in enterocytes. Therefore, a ferroxidase such as Heph or CP is necessary for ferrous iron to be oxidized to ferric iron, and it is Fpn1 that works synergistically with Heph and/or CP to mediate iron (Fe^2+^) transport across the abluminal membrane of the BBB. In short, Fpn1/Heph and/or Fpn1/CP pathways are the major mechanisms in step 2 (refs. ^18,21,40,83,84,86–94^). After crossing the abluminal membrane of BBB cells via the Fpn1/Heph and/or Fpn1/CP pathways, ferric iron could be carried away by Tf^11,12,18,31,40,81,83,95^. In addition to Tf, ferric iron might also be able to be taken away by lactotransferrin, melanotransferrin, citrate, albumin, and other carriers^81,83,96^ as Tf in cerebrospinal fluid (CSF) and interstitial fluid (IF) is fully saturated with iron and excess iron should bind to other transporters^10,31,85,97,98^.

## Expression and function of ferroportin 1, hephaestin and ceruloplasmin in the brain

The finding of Fpn1, Heph, and CP expression in the brain, including in brain capillary endothelial cells, and the related functional studies provide a foundation and solid support for the role of Fpn1/Heph and Fpn1/CP pathways in iron transport across the abluminal membrane of the BBB. In addition to capillary endothelial cells, Fpn1 is found to be widely expressed in other brain cell types, such as neurons, astrocytes, microglia, and oligodendrocytes, suggesting that Fpn1 may also be involved in iron efflux in these cells.

### Ferroportin 1

In the brain of rats, Burdo et al.^99^ were the first to demonstrate that Fpn1 is expressed in most brain regions. Jiang et al.^100^ reported that Fpn1 protein was present in the substantia nigra, cortex, striatum, and hippocampus in male Sprague–Dawley rats and found that the expression of Fpn1 protein in these brain regions was significantly affected by age. The expression of Fpn1 mRNA and/or protein has also been demonstrated in rat brain microvessels^101^, hippocampus, and cerebral cortex^102–104^. At the cellular level, Fpn1 has been shown to be expressed in neurons throughout the CNS^105^, in the endothelial cells of the developing rat brain^82^, in astrocytes, microglia, oligodendrocytes, and neurons of rats^106,107^, in primary mesencephalic cultures^108^, rat brain capillaries, and isolated rat brain endothelial cells^90^, and in primary rat brain capillary endothelial cells, pericytes, astrocytes, and neurons^109^.

In the CNS of mice, Jeong and David^110^ found that Fpn1 mRNA and protein were expressed in astrocytes and colocalized with GPI-CP (GPI-anchored CP) on the surface of purified astrocytes. Wu et al.^111^ demonstrated the expression of Fpn1 in BBB endothelial cells, neurons, astrocytes, oligodendrocytes, ependymal cells, and choroid plexus epithelial cells. Boserup et al.^112^ reported that Fpn1 was expressed in the neuronal bodies and peripheral processes of brain stem, cortex, hippocampus, thalamus, and cerebellum of the mice. You et al.^113^ showed that astrocyte hepcidin knockdown increases the expression of Fpn1 of BMVECs and that astrocyte-derived hepcidin can diminish iron uptake at the BBB from circulation through directly regulating Fpn1 in BMVECs. Chen et al.^114^ found that in mice, Fpn1 expression was positively correlated with CP expression in astrocytes and with Heph expression in oligodendrocytes. In addition, it has also been reported that Fpn1 is expressed in the oligodendrocytes of mature mice^115^, the frontal cortex of double transgenic mice (APP/PS1) at 6 and 12 months of age^116^, the hippocampus of Alzheimer disease transgenic mouse model^117^, the retinal vascular endothelial cells and pigment epithelium in mice^118^, the oligodendrocytes of mice^119^, as well as Schwann cells isolated from postnatal (P5–P7) mouse sciatic nerve^120^.

The first evidence of Fpn1 expression in the human brain was reported by Connor et al.^121^ in 2004. They demonstrated that Fpn1 expression is significantly lower in the neuromelanin cells of the substantia nigra from patients with restless legs syndrome. The immunocytochemical evidence provided by Clardy et al.^122^ showed that Fpn1 is expressed in epithelial cells of human choroid plexus and ventricular wall ependymal cells. Raha et al.^123^ reported that Fpn1 and hepcidin were widely distributed in the normal human brain and correlated with heme-positive particle deposition in the damaged vascular regions in the Alzheimer disease brain. Yanase et al.^124^ observed immunoreactivity of Fpn1 and hepcidin in the intracytoplasmic granular structures of reactive astrocytes and choroid plexus epithelial cells in autopsied human brains. Furthermore, the presence of Fpn1 has also been found in human Purkinje cell bodies and their dendrites and axons^125^, human cortical tissues and primary neurons^126^, choroid plexus obtained from human autopsy in a control group with normal neural function^127^, and human BMVECs (hBMVECs) of BBB^83,87,128^.

### Hephaestin

**Fig. 2:**
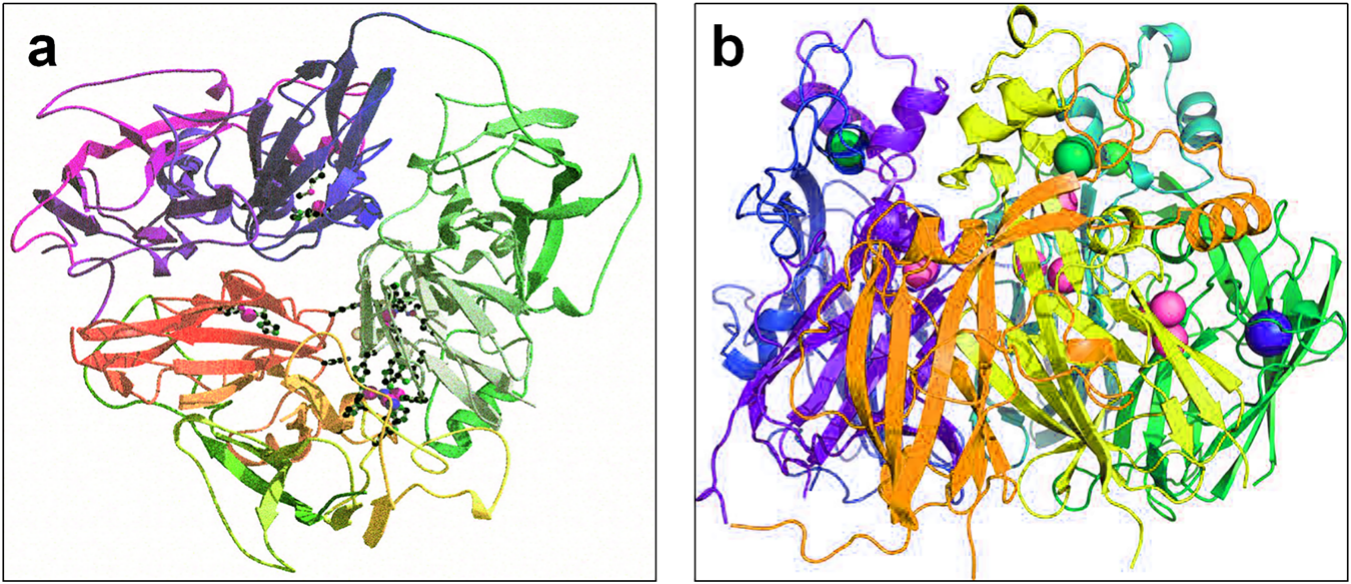
The structure of hephaestin and ceruloplasmin. **a**, The ribbon diagram of human hephaestin — top view of human hephaestin along the pseudo-threefold axis. The sequence is shaded as green for domain 1, red for domain 2, carmine for domain 3, blue for domain 4, black for domain 5, and cyan for domain 6 (ref. ^131^). **b**, The overall organization of the ceruloplasmin molecule, showing the six cupredoxin domains (domains 1, 2, 3, 4, 5, and 6 in green, cyan, blue, purple, orange, and yellow, respectively) and the locations of the metal-binding sites^137^.

Heph, encoding a transmembrane-bound CP homolog, was first described by Vulpe et al. in 1999 (ref. ^78^). Using a genetic approach, they identified this novel gene that is highly expressed in the intestine and mutant in the sex-linked anaemia (sla) mouse. Mice carrying sla mutations have impaired intestinal iron transport and develop moderate-to-severe microcytic hypochromic anemia. Heph protein, a member of the ferroxase protein family necessary for iron entry into the circulation from intestinal epithelial cells^78^, consists of an extracellular domain (which shares 50% identity with CP) attached to a single transmembrane domain and a short cytoplasmic tail^129^. The gene is located in the Xq12 region on the X chromosome^130^, and the protein is composed of 1,135 amino acids with a total molecular weight of 130.4 kDa (ref. ^131^) (Fig. 2a).

The investigation of Heph expression in the brain demonstrated that of the cortex, hippocampus, striatum, and substantia nigra of rats, all four regions have the ability to express Heph mRNA and protein and that expression could be regulated by developmental factors and iron at the transcriptional level in these brain regions^132^. Expression of Heph was also reported in the cerebral cortex and caudate putamen of mice^86^, in the cortex, hippocampus, brainstem, and cerebellum in global CP knockout (KO) and wild-type male mice^88^, Heph/CP double KO mice^92^, as well as in primary hippocampal neurons^133^, oligodendrocytes^134^, and reactive astrocytes^124^ in autopsied human brains.

Yang et al.^82^ examined the expression of ferroportin in the developing rat brain by immunoassays and RT–PCR, providing direct evidence for the expression of Heph and MTP-1 (Fpn1) in brain capillary endothelial cells. McCarthy and Kosman^83^ demonstrated the expression of Heph (as well as Fpn1, soluble CP) proteins in hBMVEC line by immunoblot and indirect immunofluorescence, also highlighting a key role of Heph, Fpn1, and sCP in iron efflux from hBMVECs. Burkhart et al.^90^ confirmed by immunocytochemistry that Heph, Fpn1, and GPI-CP as well as TfR1, ferrireductases Steap 2 and 3, and divalent metal transporter 1 were all expressed in isolated brain capillaries in vivo and in brain endothelial cells (RBECs) of rats and in immortalized rat brain endothelial (RBE4) cells in vitro.

It has been documented that mice with mutations in the Heph gene (sla mice) or double mutations in Heph (and CP) genes can induce an increase in iron accumulation in oligodendrocytes in gray matter and/or white matter^119^. In addition, mutation of Heph (and/or CP) in mice can result in iron accumulation in astrocytes and oligodendrocytes^114^ and in brain regions with oxidative damage and learning and memory defects^88,92^. Furthermore, studies on the effect of endogenous Heph (and CP) depletion (via copper chelation) on ^59^Fe efflux from hMVECs have also confirmed that the action of Heph and CP is required for iron efflux from hBMVEC Fpn1 (ref. ^83^). All these data indicate that Heph (and CP) has a role in iron efflux from brain cells, including BBB cells^83,114,133^.

### Ceruloplasmin

Holmberg and Laurell^135^ first isolated CP (the sky-blue protein) from pig serum in 1948. The CP polypeptide gene is located on chromosome 3q25. This abundant serum alpha-2 glycoprotein^136^ has a molecular weight of ~132 kDa, consists of 1,046 amino acid residues, and belongs to a family of multinuclear “blue” copper oxidases. The X-ray structure of human serum CP has been resolved at a resolution of 3.1 A, and the molecular structure comprises six plastocyanin-type domains arranged in a triangular array^137^ (Fig. 2b). This protein is mainly synthesized in hepatocytes and also in the brain^138^. Accumulating data also show that GPI-CP is a predominant form in the brain^139–141^ and indicate that GPI-CP and serum CP are highly similar and may differ only in the extreme C-terminal segment^139^. Supporting this point, CP from the circulation does not appear to cross the BBB, and soluble CP levels in CSF are extremely low, below 1 μg/ml, compared with 300–400 g/ml in plasma^139^.

In 1988, Mollgard et al. investigated the distribution and possible origin of plasma proteins in the human embryonic and fetal brain at different stages of development, localizing CP expression to astrocytes and neurons in the brain. Gitlin and co-workers^142,143^ revealed abundant CP gene expression in a specific population of astrocytes in the brain. CP has also been identified in the cortex, cerebellum, midbrain, and the eye^142^, the cortex, hippocampus, striatum, and substantia nigra of rats^144^, and age and iron status have been shown to have a significant effect on the expression of CP^144^. At the cellular level, CP is expressed in pia-arachnoid epithelial cells, hBMVECs, and ependymal cells of the choroid plexus^83,142^. In addition, it has been well confirmed that CP is recognized in distinct subsets of glial cells that primarily surround microvessels, not all astrocytes^18,83,142,143^, and positively correlated with Fpn1 expression in mouse astrocytes^114^. The CP located on these astrocytes is ideally positioned to efficiently oxidize the highly toxic ferrous iron to the ferric form and to limit ferrous iron concentrations in the CSF and IF. Astrocytes in this region have been shown to contribute directly to the BBB. This unique location implies that CP may be required for ferrous iron (Fe^2+^, the transport form of iron across the abluminal membrane of BBB) to be oxidized to ferric iron (Fe^3+^) after crossing the abluminal membrane of BBB. The latter can then bind to transport carriers in CSF and IF and be acquired by neurons, microglia, or other relevant brain cells^28,60^. Presently, the ability of brain CP to regulate iron efflux efficiency, stabilize Fpn1 membrane expression, function as ferroxidase, and regulate the oxidation of Fe^2+^ to Fe^3+^ has been well established^19,145–147^.

## Role of amyloid-β precursor protein (APP) in iron transport across the abluminal membrane of the BBB

Recent studies suggest that amyloid-β precursor protein (APP) may also have an important role in step 2. This line of research began with a study by Duce et al. in 2010 (ref. ^126^). They identified APP as a functional ferroxidase similar to CP, which can convert Fe^2+^ to Fe^3+^ after Fpn1 translocates Fe^2+^ from the cytoplasm to the plasma membrane. Both full-length APP and soluble APP (sAPP) were found to have important interactions with Fpn1 to promote iron export in cells including primary neurons. They reported that the ferroxidase center of APP is located within the REXXE consensus motif of the E2 domain, with a remote potentiation domain within the growth factor domain of E1.

However, Ebrahimi et al.^148,149^ reported shortly thereafter that APP does not catalyze iron oxide, has no ferroxidase activity in the context of non-enzymatic iron (II) oxidation by molecular oxygen, and has no ferroxidase site in its E2 domain. McCarthy et al.^128^ demonstrated that the ability of sAPP to stimulate iron efflux is due to its stabilization of Fpn1 in the plasma membrane of hBMVECs and not due to its ferroxidase activity. sAPP is able to bind to and stabilize Fpn1 on the hBMVEC cell membrane, leading to an increase in Fpn1 on the cell membrane and Heph and/or CP-dependent iron efflux^19,58,128^.

Subsequently, Ji et al.^133^ reported reduced iron efflux and increased iron accumulation in Heph KO neurons, whereas inhibition of endogenous APP by RNAi knockdown did not affect surface Fpn1 stability or iron efflux, implying that APP is not required for Fpn1-supported iron efflux in primary hippocampal neurons. Dlouhy et al.^150^ found no evidence of a comparable association between Fpn1 and full-length APP in HEK293 cells and showed that membrane Fpn1 and Heph complexes are associated with iron efflux, whereas membrane Fpn1 and APP complexes are not, suggesting that at least in HEK293 cells, APP is unnecessary for Fpn1-dependent iron efflux. A recent study^151^ demonstrated through genetic and pharmacological approaches that endocytotic amyloidogenic process of APP impairs iron output by destabilizing Fpn1 at the cell surface, whereas preferential non-amyloidogenic processes of APP at the cell surface promote Fpn1 stabilization, thereby reducing iron in neurons. From the studies and relevant discussion mentioned earlier^152–154^, it is clear that the role of APP (including full-length and sAPP, APLP1, and APLP2), in step 2 of iron transport across the BBB, requires further investigation. In addition, it is also worth investigating whether the role of APP in Fpn1 stabilization and iron efflux is cell-specific.

## Conclusion and perspectives

**Fig. 3:**
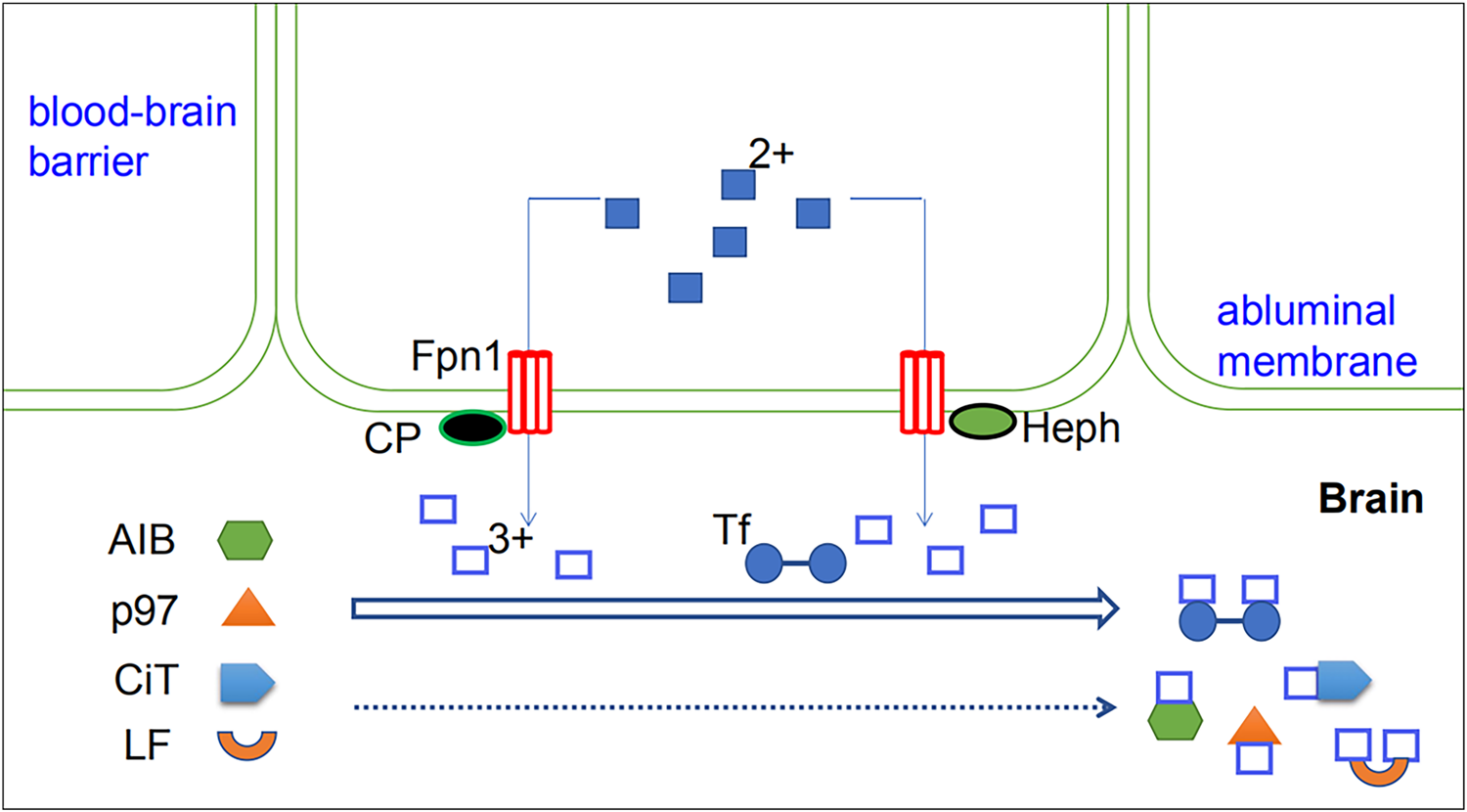
Ferroportin 1 (Fpn1) is a key player in iron transport across the abluminal membrane of the blood–brain barrier. Iron, in the form of ferrous iron (Fe^2+^), is first mediated by Fpn1 transport across the abluminal membrane of the BBB, and Fe^2+^ on the surface of abluminal membrane is subsequently oxidized by ceruloplasmin (CP) or hephaestin (Heph) to Fe^3+^, which is eventually carried away by Tf and/or other carriers (including LF (lactotransferrin), p97 (melanotransferrin), CiT (citrate), and AlB (albumin)) and enters the brain.

On the basis of the findings reviewed in the preceding section, we can conclude that Fpn1 is a key player in iron transport across the abluminal membrane of the BBB. The Fpn1/Heph and/or Fpn1/CP iron export pathways found in the basal membrane of intestinal epithelial cells in the gut have the same role in Fe^2+^ transport across the abluminal membrane of the BBB and possibly also at the abluminal membrane of the blood retinal barrier^94,155^. Iron (Fe^2+^) in the form of ferrous iron is first transported by Fpn1 across the abluminal membrane of BBB, and Fe^2+^ on the surface of abluminal membrane is subsequently oxidized to Fe^3+^ by CP or Heph, which is eventually taken away by Tf and/or other carriers and enters the brain (Fig. 3). The aforementioned description of the transport process of iron across the abluminal membrane of the BBB is well verified and widely accepted.

However, a number of highly relevant issues regarding this process have yet to be elaborated. First, it remains unclear that under physiological conditions, which of the Fpn1/Heph and Fpn1/CP pathways has a dominant role, or what the functional relationship is between these two pathways in the process of iron transport across the abluminal membrane of the BBB. On the basis of the current understanding of where Heph and CP are expressed in the brain, it is reasonable to assume that the role of the two pathways, Fpn1/Heph and Fpn1/CP, may be location-dependent. The pn1/CP pathway may have a dominant role in the abluminal membrane of the BBB that surrounds astrocytes, because CP is expressed in distinct subsets of glial cells that predominantly surround microvessels, rather than all astrocytes^18,83,142,143^, whereas the Fpn1/Heph pathway may have a dominant role in other parts of the BBB because Heph is widely expressed in the brain^78,82,86,88,124,132–134^.

Second, it must be pointed out that the scope of current discussion and analysis relies primarily on in vitro and in vivo studies, which is one of the major limitations of this Review. At present, relevant findings that can be drawn from human data is still very limited. Therefore, detailed and precise clinical and human studies are absolutely needed to conclusively confirm the role of Fpn1, as well as Heph and CP, in the process of iron efflux from BBB cells. It should also be noted that the currently available data are insufficient to reject a role for APP in ferrous iron transport across the abluminal membrane of the BBB. Although the evidence against such a role outweighs the evidence for, further research on this issue is absolutely needed.

Third, it is critically important to elucidate the relevant factors and mechanisms that control the process of iron efflux and the expression of Fpn1, Heph, and CP on the abluminal membrane of the BBB. Recent studies suggest that this process could be influenced by astrocyte-secreted agents, hBMVEC-secreted cytokine IL-1b and IL-6 (refs. ^18,19,58,87,156^), tumor necrosis factor-alpha^157^, dynamic iron requirements of the neurovascular unit^19^, apo-Tf and deferoxamine^21^, iron chelator (PBT434)^158^, apo-Tf and holo-Tf regulation^93^, and by IL-6 and calcium^159^. However, further systematic and detailed research on this issue is needed.

Fourth, there is also a need to investigate whether zyklopen (or other ferrioxide enzymes and CP homologs), which has been shown to be highly expressed in the choroid plexus of the mouse brain^160^, is involved in this process^19^. Clarifying these key questions will greatly improve our understanding of brain iron metabolism and the role of iron in neurodegenerative diseases and provide more useful information for harnessing the brain iron delivery system to deliver therapeutic drugs for the treatment of many neurological diseases.

## Data Availability

Not applicable.
